# Inhibition of SARS-CoV-2 replication by a ssDNA aptamer targeting the nucleocapsid protein

**DOI:** 10.1128/spectrum.03410-23

**Published:** 2024-02-20

**Authors:** Yanping Huang, Congcong Huang, Junkai Chen, Siwei Chen, Bei Li, Jian Li, Zhixiong Jin, Qiwei Zhang, Pan Pan, Weixing Du, Long Liu, Zhixin Liu

**Affiliations:** 1 Department of Infectious Diseases, Renmin Hospital, School of Basic Medical Sciences, Hubei University of Medicine, Shiyan, China; 2 Institute of Virology, Virology Key Laboratory of Shiyan City, Shiyan, China; 3 Hubei Key Laboratory of Embryonic Stem Cell Research, Hubei University of Medicine, Shiyan, China; 4 Guangdong Provincial Key Laboratory of Virology, Institute of Medical Microbiology, Jinan University, Guangzhou, China; 5 The First Affiliated Hospital of Jinan University, Guangzhou, China; Wright State University, Dayton, Wright, USA

**Keywords:** SARS-CoV-2, nucleocapsid protein, aptamer, antiviral therapy

## Abstract

The nucleocapsid protein of SARS-CoV-2 plays significant roles in viral assembly, immune evasion, and viral stability. Due to its immunogenicity, high expression levels during COVID-19, and conservation across viral strains, it represents an attractive target for antiviral treatment. In this study, we identified and characterized a single-stranded DNA aptamer, N-Apt17, which effectively disrupts the liquid-liquid phase separation (LLPS) mediated by the N protein. To enhance the aptamer’s stability, a circular bivalent form, cb-N-Apt17, was designed and evaluated. Our findings demonstrated that cb-N-Apt17 exhibited improved stability, enhanced binding affinity, and superior inhibition of N protein LLPS; thus, it has the potential inhibition ability on viral replication. These results provide valuable evidence supporting the potential of cb-N-Apt17 as a promising candidate for the development of antiviral therapies against COVID-19.

**IMPORTANCE:**

Variants of SARS-CoV-2 pose a significant challenge to currently available COVID-19 vaccines and therapies due to the rapid epitope changes observed in the viral spike protein. However, the nucleocapsid (N) protein of SARS-CoV-2, a highly conserved structural protein, offers promising potential as a target for inhibiting viral replication. The N protein forms complexes with genomic RNA, interacts with other viral structural proteins during virion assembly, and plays a critical role in evading host innate immunity by impairing interferon production during viral infection. In this investigation, we discovered a single-stranded DNA aptamer, designated as N-Apt17, exhibiting remarkable affinity and specificity for the N protein. Notably, N-Apt17 disrupts the liquid-liquid phase separation (LLPS) of the N protein. To enhance the stability and molecular recognition capabilities of N-Apt17, we designed a circular bivalent DNA aptamer termed cb-N-Apt17. In both *in vivo* and *in vitro* experiments, cb-N-Apt17 exhibited increased stability, enhanced binding affinity, and superior LLPS disrupting ability. Thus, our study provides essential proof-of-principle evidence supporting the further development of cb-N-Apt17 as a therapeutic candidate for COVID-19.

## INTRODUCTION

SARS-CoV-2, a member of the beta coronavirus genus, possesses an unsegmented single-stranded positive-strand RNA genome (1). It is characterized by four structural proteins: spike (S), envelope (E), membrane (M), and nucleocapsid (N) (2–4). Among these proteins, the nucleocapsid (N) protein is notably abundant and highly conserved across coronaviruses (5).

The SARS-CoV-2 N protein consists of 419 amino acids and is composed of an N-terminal domain and a C-terminal domain. It also features three irregular flexible regions that serve as connectors between the two domains and two sides (6). The N protein plays a crucial role in recognizing and packaging viral RNA into ribonucleoprotein complexes. Moreover, it participates in various processes such as viral transcription, replication, and immune regulation through its interactions with viral and host proteins (7–10). Notably, the N protein elicits both humoral and cellular immune responses upon infection, making it an important target for vaccine development (11).

The SARS-CoV-2 N protein interacts with viral genomic RNA and undergoes liquid-liquid phase separation (LLPS) (12). LLPS is a phenomenon in which proteins and nucleic acids condense into liquid-like droplet structures (13). During viral infection, LLPS initiates viral replication and facilitates the assembly of viral structural proteins (13, 14). Thus, the LLPS induced by the SARS-CoV-2 N protein plays a critical role in viral RNA packaging, modulation of stress granules, and regulation of the host cell’s antiviral innate immune pathway.

In this study, we selected a high-affinity single-stranded DNA (ssDNA) aptamer that disrupts the LLPS of the N protein, leading to the inhibition of viral replication. Therefore, our findings provide a diagnostic and therapeutic candidate for the treatment of COVID-19.

## RESULTS

### Enrichment and identification of DNA aptamers against SARS-CoV-2 N protein

**Fig 1:**
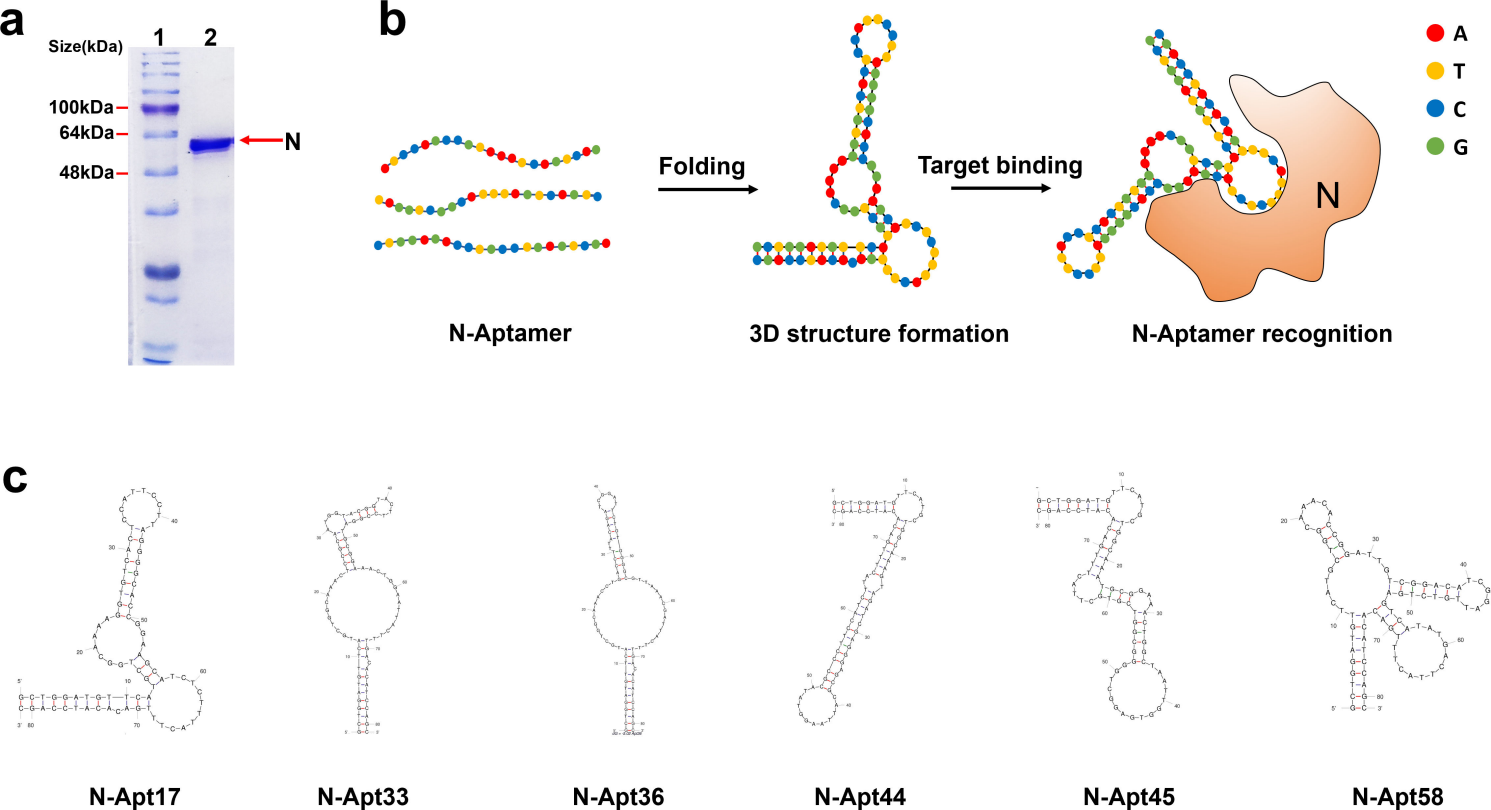
Screening of ssDNA aptamers targeting the recombinant SARS-CoV-2 N protein. (**a**) Purified recombinant SARS-CoV-2 N-His_6_ protein. Lane 1: protein ladder; lane 2: SDS-PAGE analysis of the purified N-His_6_ protein. (**b**) Schematic diagram illustrating the recognition of N protein by ssDNA aptamers. (**c**) Predicted secondary structures of the N-aptamers (N-Apt17, N-Apt33, N-Apt36, N-Apt44, N-Apt45, N-Apt58, and N-Apt17) that specifically recognize the SARS-CoV-2 N protein, as determined by Mfold software analysis.

In this study, our primary objective was to enrich and identify DNA aptamers capable of specifically binding to the SARS-CoV-2 N protein. We employed the Systematic Evolution of Ligands by Exponential Enrichment (SELEX) method to achieve this (15). First, we expressed and purified the SARS-CoV-2 N protein using a prokaryotic expression system (Fig. 1a). This ensured a sufficient quantity of the target protein for subsequent experiments. Next, we performed multiple rounds of SELEX using a diverse library of DNA oligonucleotides. Each round involved incubating the N protein with the DNA library, followed by separation of the protein-DNA complexes from the unbound DNA. The bound DNA was then eluted and amplified by PCR to generate a new pool of DNA sequences for the subsequent round. After several rounds of SELEX, we obtained a pool of DNA aptamers that exhibit binding ability toward the SARS-CoV-2 N protein. To identify the individual aptamers, we performed cloning and sequencing of the selected DNA molecules. As a result of our investigation, six high-affinity aptamers, named N-Apt17, N-Apt33, N-Apt36, N-Apt44, N-Apt45, and N-Apt58, were identified, demonstrating binding to the SARS-CoV-2 N protein (Fig. 1b). To gain insights into the secondary structures of these aptamers, we employed the Mfold software (16), which predicted the folding patterns based on their specific base sequences (Fig. 1c).

Overall, we have successfully enriched and identified a panel of DNA aptamers that exhibit binding ability toward the SARS-CoV-2 N protein. These aptamers hold potential for further investigation and development as diagnostic or therapeutic agents against COVID-19.

### N protein binding affinity and specificity of six N-aptamers

**Fig 2:**
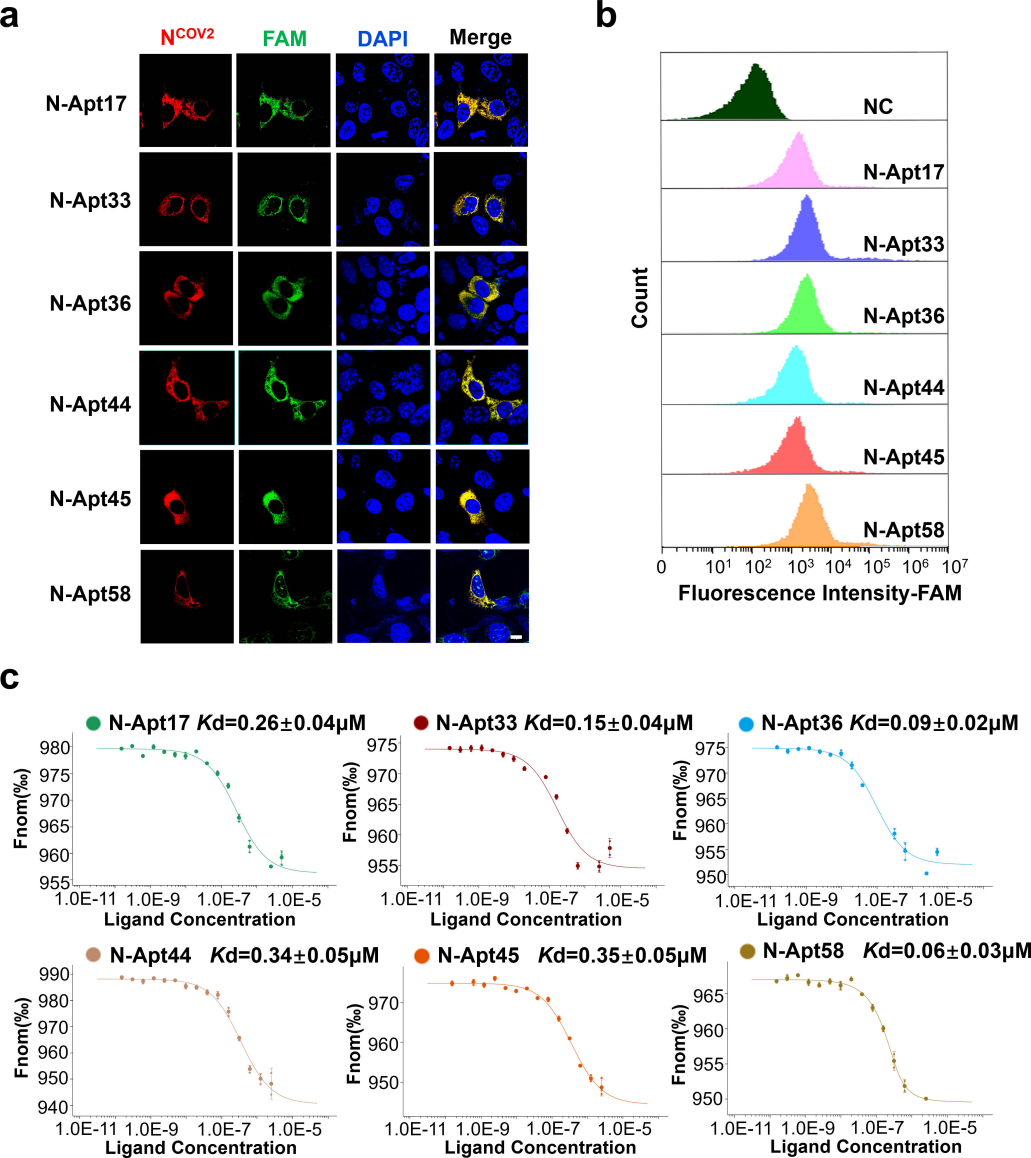
Affinity and specificity of six N-aptamers binding to N protein. (**a**) Confocal microscopy images showing the colocalization of N protein and FAM-labeled N-aptamers in HEK293T cells overexpressing N protein. Scale bars: 10 µm. (**b**) Flow cytometry analysis of FAM-labeled N-aptamers and a negative control (NC) (random sequence) co-incubated with N protein immobilized on magnetic beads. (**c**) Measurement of the *Kd* (dissociation constant) value between N-aptamers and N protein using MST-based assay.

To evaluate the binding affinity and specificity of the six identified N-aptamers (N-Apt17, N-Apt33, N-Apt36, N-Apt44, N-Apt45, and N-Apt58) for the SARS-CoV-2 N protein, we conducted a series of experiments. Initially, FAM-labeled N-aptamers were transfected into HEK293T cells overexpressing N-RFP (Red Fluorescent Protein, RFP). Fluorescence analysis revealed the colocalization of the N protein with all six N-aptamers within the cytoplasm, substantiating their binding interaction (Fig. 2a). Furthermore, noteworthy observations indicated that N-Apt58 exhibited nuclear localization, suggesting potential cytotoxic effects on cells.

Next, we employed anti-His_6_ magnetic beads to immobilize purified N-His_6_ protein. Subsequently, we incubated the immobilized N protein with FAM-labeled N-aptamers and performed flow cytometry analysis. The results confirmed that all six N-aptamers exhibited specific binding ability with the N-His_6_ protein (Fig. 2b). To quantify the binding affinity between the N protein and the N-aptamers, we determined their dissociation constants (*Kd* values) using Microscale Thermophoresis (MST). The results demonstrated that the *Kd* values of the N protein and the six N-aptamers ranged from 0.35 ± 0.04 µM to 0.06 ± 0.03 µM, indicating strong binding affinity (Fig. 2c).

Overall, these findings indicate that the six N-aptamers possess high binding affinity and specificity toward the SARS-CoV-2 N protein.

### N-Apt17 inhibits N protein LLPS

To investigate whether the identified N-aptamers could inhibit the LLPS of the N protein, we conducted several experiments.

**Fig 3:**
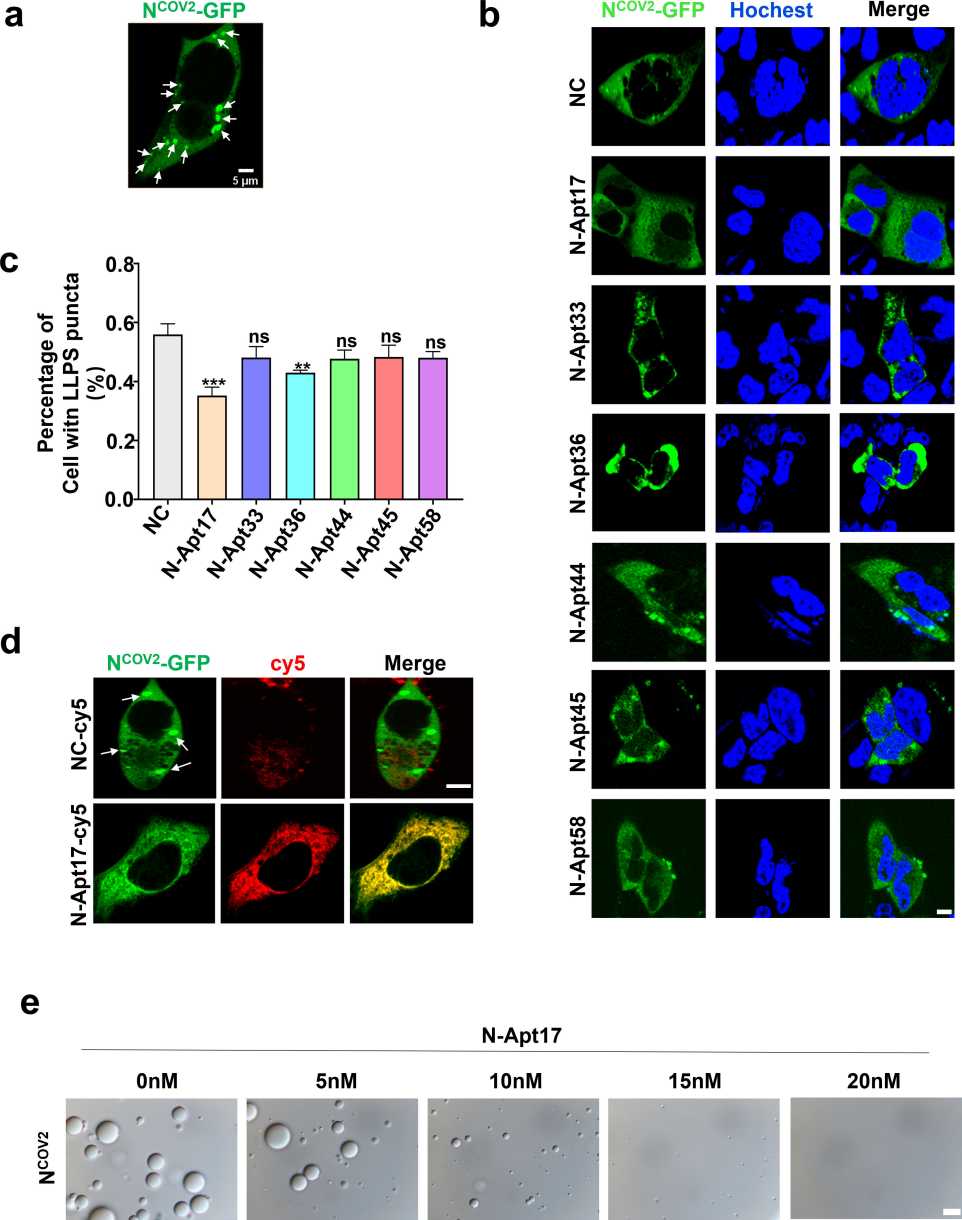
N Apt17 inhibits N protein LLPS. (**a**) Microscopic images showing LLPS produced by N-GFP protein in HEK293T cells (white arrow). Scale bars: 5 µm. (**b**) Confocal microscopy images of N-GFP overexpressing HEK293T cells transfected with six different N-aptamers, showing the effect on N protein LLPS. Scale bars: 5 µm. (**c**) Statistical analysis of the inhibitory effect of different N-aptamers on N protein LLPS. ns, no significant difference; **P < 0.01; ***P < 0.001. (**d**) Confocal microscopy images demonstrating the inhibitory effect of N-Apt17-cy5 on N protein LLPS. White arrows indicate the N-GFP LLPS in the cell. Scale bars: 5 µm. (**e**) Observation of the inhibitory effect of N-Apt17 at different concentrations on N protein LLPS *in vitro*. Scale bars: 10 µm.

First, we overexpressed N-GFP in HEK293T cells and observed the formation of LLPS in the cytoplasm (Fig. 3a). Subsequently, we transfected the N-aptamers into the N-GFP overexpressed cells and evaluated the proportion of cells exhibiting LLPS formation in different N-aptamer treatment groups using fluorescence microscopy. Interestingly, we found that N-Apt17 demonstrated the most significant inhibition of LLPS formation (Fig. 3b and c).

To further investigate the inhibitory effect of N-Apt17 on N protein LLPS, we transfected cy5-labeled N-Apt17 into N-GFP overexpressed HEK293T cells and observed the cells using confocal microscopy. The results clearly showed that N-Apt17 effectively inhibited N-GFP LLPS formation (Fig. 3d).

Furthermore, we observed LLPS formation of purified N-His_6_ *in vitro* using differential interference contrast (DIC) microscopy. We then added different concentrations of N-Apt17 into the solution to assess its inhibitory effect on N-His_6_ LLPS. The results revealed that N-Apt17 exhibited LLPS inhibition ability at very low concentrations (5 nM) and completely abolished N-His_6_ LLPS at a concentration of 20 nM (Fig. 3e).

These findings demonstrate that among the six identified N-aptamers, N-Apt17 displayed the most significant inhibition of N protein LLPS. N-Apt36 also exhibited a certain degree of inhibitory effect, while the other four N-aptamers showed limited inhibition ability. However, further investigation is required to determine whether these N-aptamers can block other biological functions of the N protein.

### Stability analysis of N-Apt17 and cb-N-Apt17

In an effort to improve the stability of N-Apt17, we designed a circular bivalent aptamer termed cb-N-Apt17. The rationale behind this design is to leverage the circular structure of cb-N-Apt17, aiming to confer resistance to exonuclease degradation while preserving its specificity and affinity.

**Fig 4:**
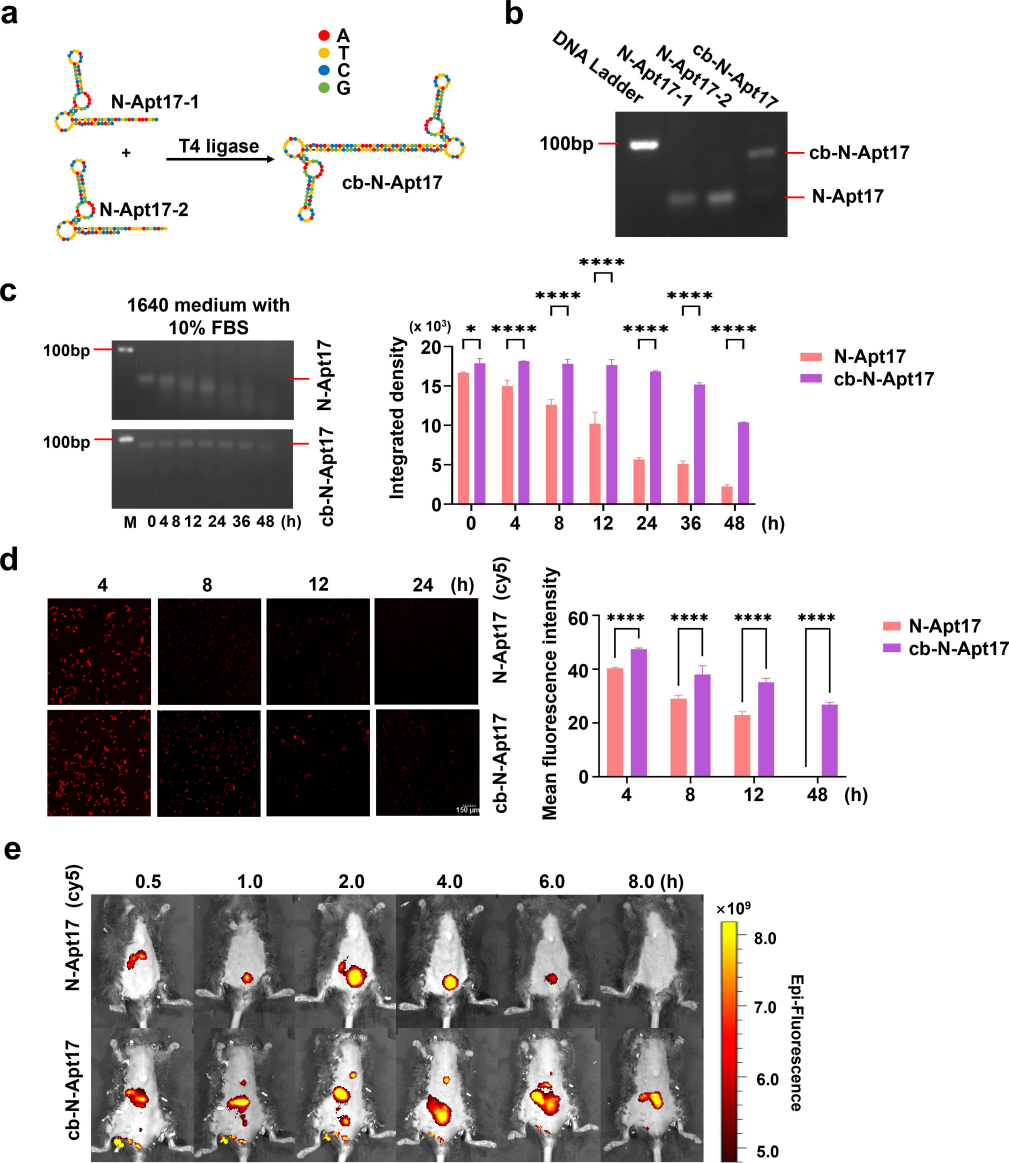
Stability analysis of N-Apt17 and cb-N-Apt17. (**a**) Schematic diagram illustrating the construction process and structure of cb-N-Apt17. (**b**) Agarose electrophoresis analysis of N-Apt17 and cb-N-Apt17. (**c**) Determination of the stability of N-Apt17 and cb-N-Apt17 by agarose electrophoresis after incubation for different periods in 1640 medium supplemented with 10% FBS (left). Quantification of band intensity for N-Apt17 and cb-N-Apt17 using Image J software and calculation using GraphPad software (right). M, DNA marker. (**d**) Observation of intracellular degradation rate of N-Apt17-cy5 and cb-N-Apt17-cy5 in HEK293T cells using fluorescence microscopy at different time points (left). Quantification of fluorescence signaling with Image J software and calculation using GraphPad software (right). (**e**) *In vivo* detection of the signal of N-Apt17-cy5 and cb-N-Apt17-cy5 in mice after intravenous injection via tail vein, assessed by *in vivo* bioluminescence imaging at different time points.

To construct cb-N-Apt17, two N-Apt17 strands with a 5′-phosphate group and a 3′-OH group were designed, along with additional flanking complementary sequences. These strands were linked using T4 DNA ligase (Table S1; Fig. 4a). The efficiency of cb-N-Apt17 preparation was verified by investigating the lengths of the complementary sequences using agarose electrophoresis (Fig. 4b).

The physical stability of cb-N-Apt17 was evaluated by testing its anti-degradation ability compared to N-Apt17 in RPMI 1640 medium supplemented with 10% fetal bovine serum (FBS) (Fig. 4c, left). The integrated density of different lanes, indicative of the integrity of N-Apt17 and cb-N-Apt17 at distinct time points, was meticulously analyzed using ImageJ software. The results were then graphically represented using GraphPad Prism 9 software (Fig. 4c, right). The results showed that N-Apt17 started to degrade after 4 hours of incubation with 10% FBS. In contrast, even after 36 hours of incubation with 10% FBS, the integrity of the cb-N-Apt17 band was still evident.

To evaluate the enhanced suitability of cb-N-Apt17 for *in vivo* applications, we compared the stability of Cy5-labeled N-Apt17 (N-Apt17-cy5) to that of Cy5-labeled cb-N-Apt17 (cb-N-Apt17-cy5) in HEK293T cells (Fig. 4d, left). For a comprehensive assessment, we employed ImageJ software to quantitatively analyze the mean fluorescence intensity of images at different time points, providing insights into the stability of N-Apt17 and cb-N-Apt17 in HEK293T cells. The obtained results were meticulously charted using GraphPad Prism 9 software, ensuring an objective and clear presentation of the data (Fig. 4d, right). The findings revealed that the signal of N-Apt17-cy5 diminished more rapidly, becoming almost invisible at 24 hours. In contrast, cb-N-Apt17-cy5 continued to exhibit visible signals even at the 24-hour mark, showcasing its enhanced stability over time.

It is noteworthy that through *in vivo* bioluminescence imaging, we observed that upon tail vein injection into mice, N-Apt17 rapidly appeared at the liver location of the mice (0.5 hours). Signals could be observed at the bladder position of the mice between 1 and 6 hours, but by 8 hours, the fluorescent signal was no longer detectable. In contrast, cb-N-Apt17 exhibited a slower degradation rate, with signals observable at the liver position within 0.5–2 hours and the remaining detectable at the abdominal area of the mice between 4 and 8 hours, during which no fluorescence signal was observed at the bladder position (Fig. 4e).

Overall, these results indicate that cb-N-Apt17 exhibits significantly improved physical stability compared to N-Apt17. Additionally, *in vivo* bioluminescence imaging indicates that both cb-N-Apt17 and N-Apt17 may initially accumulate in the liver and subsequently enter the bladder through metabolism in mice. However, further investigation is needed to understand the specific distribution differences.

### cb-N-Apt17 has improved binding affinity and specificity and inhibits N protein LLPS more effectively

**Fig 5:**
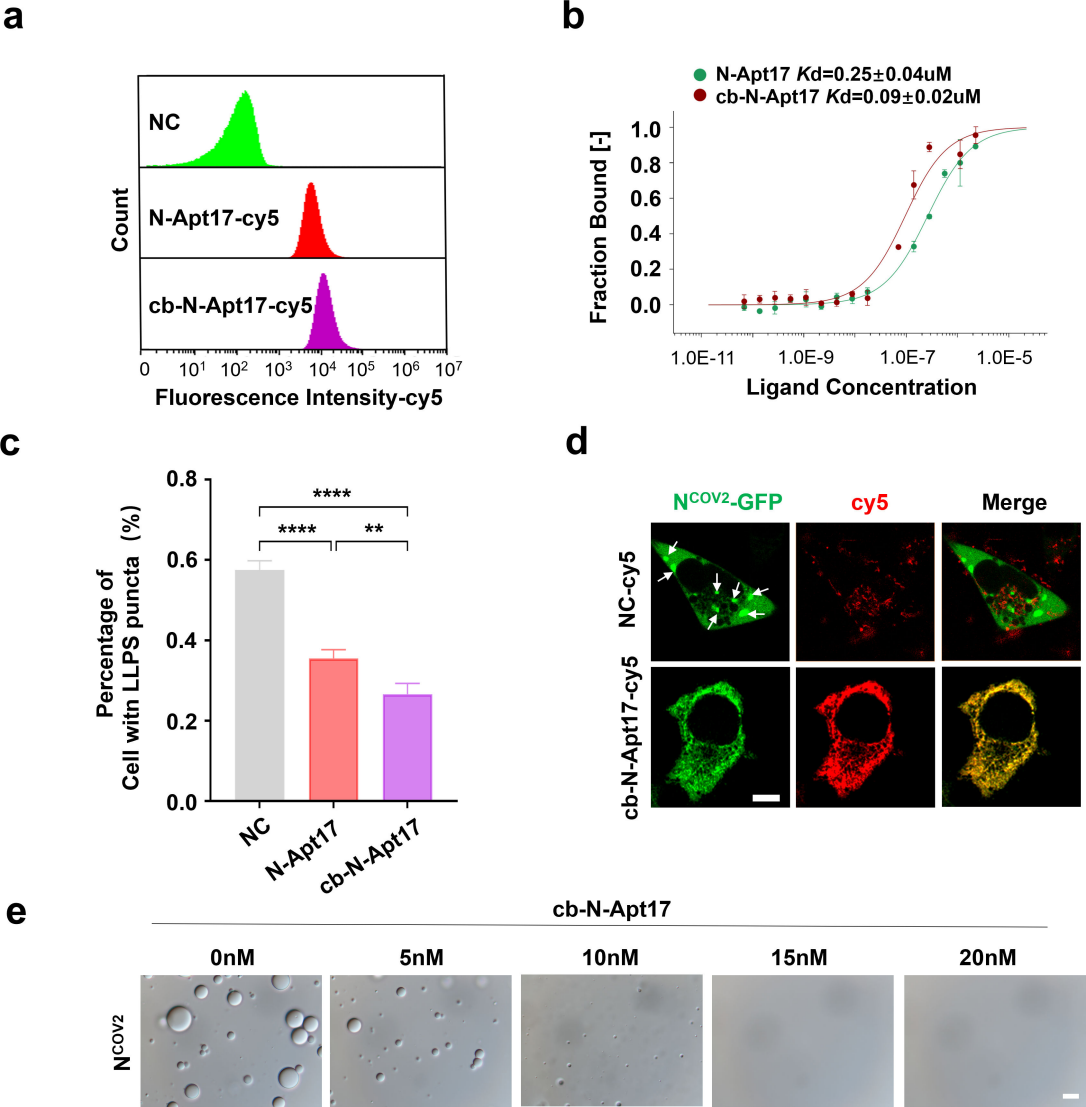
cb-N-Apt17 exhibits improved binding affinity and specificity, leading to enhanced inhibition of N protein LLPS. (**a**) Flow cytometry analysis of cy5-labeled N-Apt17, cb-N-Apt17, or negative control (NC) co-incubated with magnetic bead-fixed N protein. (**b**) Measurement of the dissociation constant (*Kd*) values between N-Apt17 or cb-N-Apt17 and N protein using MST-based assay. (**c**) Statistical analysis of the inhibitory effect of N-Apt17 and cb-N-Apt17 on N protein LLPS in N-GFP overexpressing HEK293T cells. *P < 0.05; **P < 0.01; ****P < 0.0001. (**d**) Confocal microscopy images showing the LLPS of N protein in N-GFP overexpressing HEK293T cells transfected with cb-N-Apt17 or NC. White arrows indicate N protein LLPS within the cell. Scale bars: 5 µm. (**e**) Observation of the inhibitory effect of cb-N-Apt17 at different concentrations on N protein LLPS *in vitro*. Scale bars: 10 µm.

To evaluate the binding affinity and specificity of cb-N-Apt17, experiments were conducted, comparing its performance to that of N-Apt17. Figure 5a and b illustrate that cb-N-Apt17 displayed enhanced binding ability to the N protein and superior protein affinity when compared to N-Apt17. These findings strongly suggest that cb-N-Apt17 possesses a heightened binding affinity for the N protein. Moreover, in terms of inhibiting N protein LLPS, cb-N-Apt17 was found to be more effective than N-Apt17 (Fig. 5c and d). These findings suggest that cb-N-Apt17 not only possesses a longer half-life and improved stability *in vivo* but also exhibits enhanced binding affinity and specificity, leading to more effective inhibition of N protein LLPS.

As previously report, by using DIC microscopy, we observed LLPS formation of purified N-His_6_ *in vitro*. Subsequently, we introduced varying concentrations of cb-N-Apt17 into the solution to evaluate its inhibitory impact on N protein LLPS. The findings indicated that cb-N-Apt17 demonstrated LLPS inhibition capability even at extremely low concentrations (5 nM), and at a concentration of 15 nM, it completely eliminated N protein LLPS (Fig. 5e).

Overall, these results highlight the advantages of cb-N-Apt17, as it combines improved stability with enhanced binding properties, making it a promising candidate for further development as a therapeutic agent for inhibiting N protein function in viral infection.

### Cytotoxicity test of N-Apt17 and cb-N-Apt17

**Fig 6:**
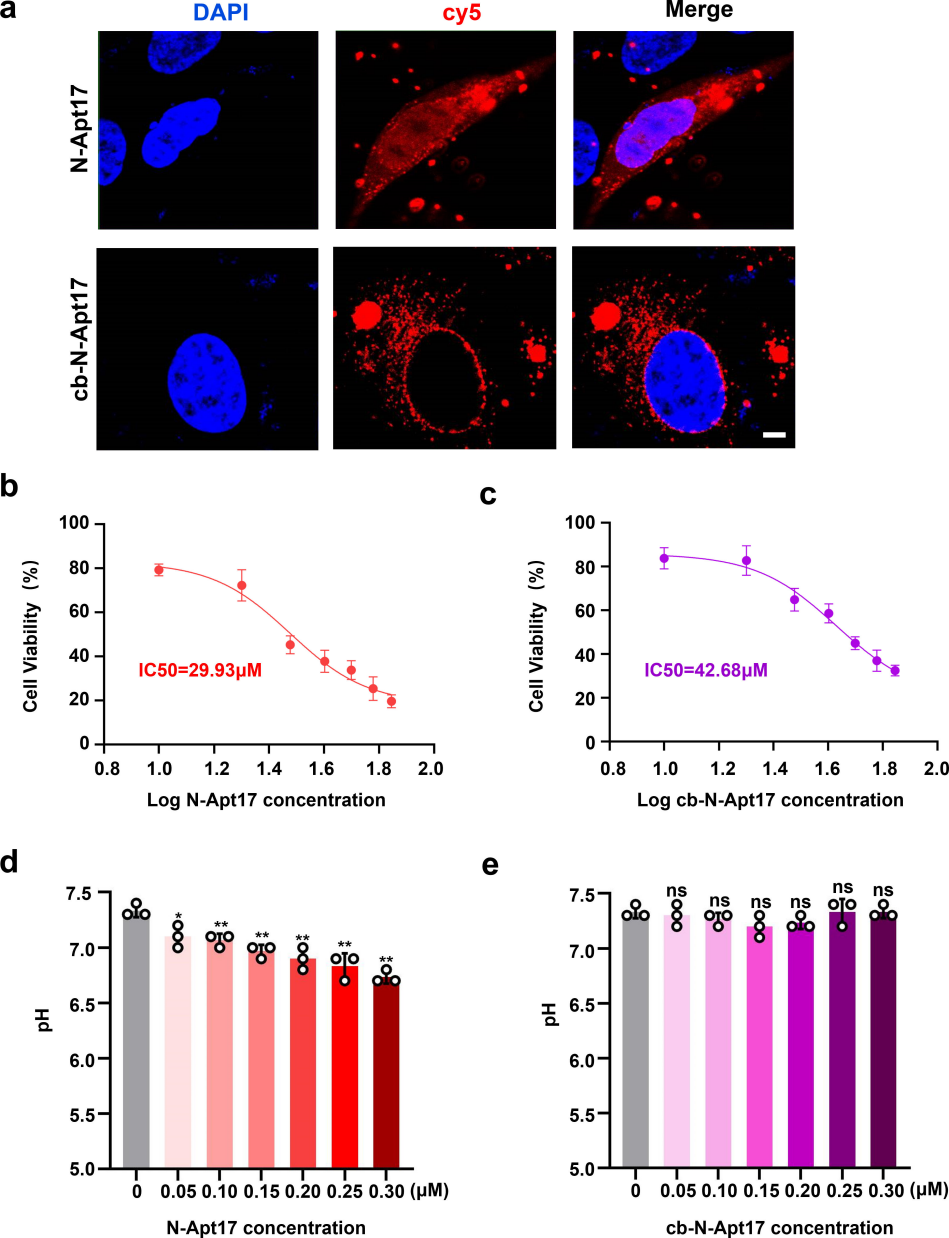
Cytotoxicity assessment of N-Apt17 and cb-N-Apt17. (a) Confocal microscopy images showing the subcellular localization of N-Apt17-cy5 and cb-N-Apt17-cy5 in transfected HEK293T cells. Scale bars: 10 µm. (b and c) CCK8 cell proliferation and viability assay used to evaluate the IC50 of N-Apt17 and cb-N-Apt17. (d and e) pH changes in the system within the concentration range for N-Apt17 or cb-N-Apt17 *in vitro* or *in vivo* application. ns, not significant difference; *P < 0.05; **P < 0.01.

Continuing, we examined the cellular localization and distribution of N-Apt17-cy5 and cb-N-Apt17-cy5 in the absence of the N protein. The findings demonstrated that cb-N-Apt17-cy5 primarily localized in the cytoplasm, whereas N-Apt17-cy5 was observed in both the cytoplasm and nucleus (Fig. 6a). This discrepancy could be attributed to the larger molecular weight of cb-N-Apt17-cy5, which likely prevents it from entering the nucleus. The previous detection of N-Apt17 distribution in the cytoplasm (Fig. 2a) was likely a result of colocalization with the N protein.

Considering the localization of N-Apt17 in the nucleus, there was a concern about its potential cytotoxicity. Therefore, a CCK-8 cell proliferation and viability assay were conducted to assess the cytotoxic effects of N-Apt17 and cb-N-Apt17. Cell viability in different wells and IC50 values for N-Apt17 and cb-N-Apt17 were statistically analyzed using GraphPad Prism 9. The IC50 for N-Apt17 was determined to be 29.93 µM (Fig. 6b), while for cb-N-Apt17, it was 42.68 µM (Fig. 6c). Given the relatively high concentrations for a small nucleic acid molecule, these results indicate that both N-Apt17 and cb-N-Apt17 exhibit minimal toxicity to cells.

To investigate whether the inhibition of N protein LLPS by the aptamers was due to pH changes in the system, we measured the pH changes in the solution within the concentration range applicable for *in vitro* or *in vivo* experiments. The results indicated that within the concentration range of 0 μM to 0.3 µM, the pH of the N-Apt17 solution experienced only a slight decrease (Fig. 6d), while the pH of the cb-N-Apt17 solution remained relatively unchanged (Fig. 6e).

These findings suggest that while the subcellular localization of N-Apt17-cy5 and cb-N-Apt17-cy5 differed, neither aptamer exhibited obvious cytotoxicity. Additionally, both aptamers did not significantly alter the pH of the system within the concentration range intended for our *in vitro* or *in vivo* application.

### cb-N-Apt17 binds to major SARS-CoV-2 N variants

To assess the binding affinity and specificity of cb-N-Apt17 with major SARS-CoV-2 N protein variants, we conducted experiments using the original strain N protein (WT-N), delta variant N protein (Delta-N), and omicron variant N protein (Omicron-N).

**Fig 7:**
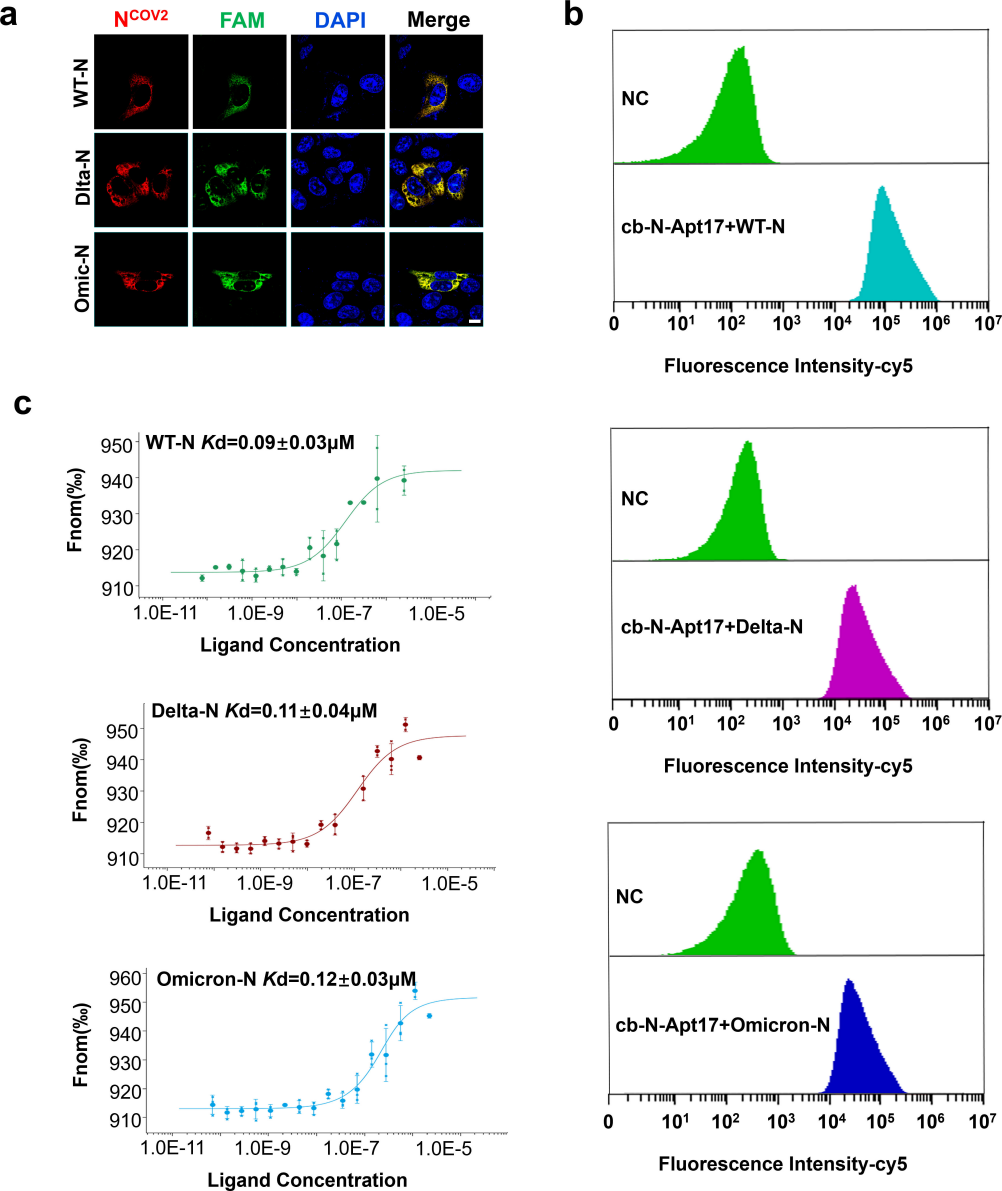
Binding ability of cb-N-Apt17 to major SARS-CoV-2 N variants. (**a**) Confocal microscopy images showing the colocalization of N protein and FAM-labeled N-aptamers in HEK293T cells overexpressing N protein. Scale bars: 10 µm. (**b**) Flow cytometry analysis of FAM-labeled N-aptamers and a negative control (NC, random sequence) co-incubated with magnetic bead-fixed N protein. (**c**) Measurement of the dissociation constant (*Kd*) value between N-aptamers and N protein using MST-based assay.

The results showed that cb-N-Apt17 exhibited colocalization with all three N proteins in the cytoplasm (Fig. 7a). To further confirm the binding specificity, we fixed the three recombinant N proteins using magnetic beads and then co-incubated them with FAM-labeled cb-N-Apt17 before performing flow cytometry analysis. The results confirmed the specific binding ability of cb-N-Apt17 with all three N proteins (Fig. 7b).

Furthermore, we determined the dissociation constants (*Kd* values) between cb-N-Apt17 and the three N proteins using MST. The results demonstrated that the *Kd* values of cb-N-Apt17 with the three N protein variants ranged from 0.09 ± 0.03 µM to 0.12 ± 0.03 µM (Fig. 7c).

These findings indicate that cb-N-Apt17 exhibits high binding affinity and specificity not only with the original strain N protein but also with major SARS-CoV-2 N protein variants, including the delta and omicron variants. This suggests that cb-N-Apt17 could be a promising therapeutic candidate for targeting multiple SARS-CoV-2 N protein variants.

### cb-N-Apt17 inhibits SARS-CoV-2 replication in Caco2 cells

To investigate the antiviral effect of cb-N-Apt17, we utilized a transcription and replication-competent SARS-CoV-2 virus-like particle (trVLP) system expressing a reporter gene (eGFP) instead of the viral nucleocapsid gene.

First, we overexpressed the major SARS-CoV-2 N protein variants in Caco2 cells. Subsequently, we transfected cb-N-Apt17 into the Caco2 cells before infecting them with SARS-CoV-2 GFP/ΔN trVLP. Then, we examined one round of replication of SARS-CoV-2 GFP/ΔN trVLP in the N protein overexpressed Caco2 cells.

**Fig 8:**
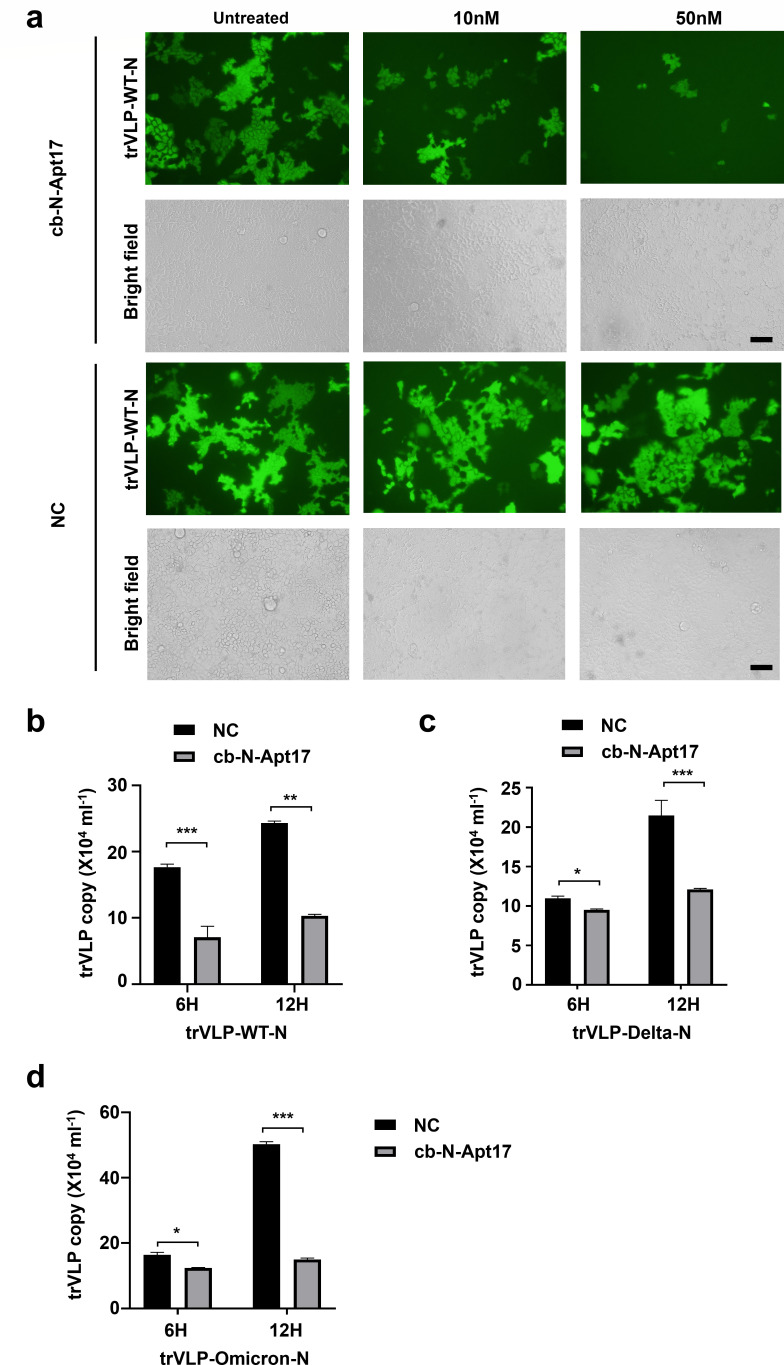
cb-N-Apt17 inhibits the replication of major SARS-CoV-2 trVLP variants. (**a**) Inhibition of trVLP-WT-N infection (0.5 MOI (Multiplicity of Infection)) by cb-N-Apt17 in Caco2 cells. Different concentrations of cb-N-Apt17 (0 nM, 10 nM, and 50 nM) were transfected into Caco2 cells that overexpressed N-WT (the original strain N protein). Subsequently, microscopic images were captured after infecting the cells with trVLP for a duration of 12 hours. Scale bars: 50 µm. (**b**) Quantification of SARS-CoV-2 trVLP copies present in the cell supernatant using QRT-PCR. The replication of trVLP-WT-N in Caco2 cells was significantly inhibited upon treatment with cb-N-Apt17 (50 nM) at both 6 and 12 hours post infection. (**c**) Similarly, replication of trVLP-Delta-N in Caco2 cells was also significantly hindered when treated with cb-N-Apt17 (50 nM) at both 6 and 12 hours post infection. (**d**) Furthermore, the replication of trVLP-Omicron-N in Caco2 cells was substantially suppressed by cb-N-Apt17 (50 nM) at both 6 and 12 hours post infection. *P < 0.05; **P < 0.01; ***P < 0.001.

Our observations revealed a decrease in eGFP signal as the concentration of cb-N-Apt17 increased (Fig. 8a; Fig. S1 and S2). Additionally, we collected the supernatant containing SARS-CoV-2 GFP/ΔN trVLPs and determined the viral titers at different time points using QRT-PCR (Quantitative Real-time PCR). Consistent with the eGFP expression results, the QRT-PCR data also demonstrated that cb-N-Apt17 inhibited the replication of SARS-CoV-2 trVLP (Fig. 8b, c, and d).

These results suggest that cb-N-Apt17 effectively inhibits the replication of various SARS-CoV-2 variants, including the original strain (WT-N) (Fig. 8a and b), delta variant (Delta-N) (Fig. 8c; Fig. S1), and omicron variant (Omicron-N) (Fig. 8d; Fig. S2). The inhibitory effect was observed both in terms of microscopic images and quantitative analysis using QRT-PCR. These findings indicate the potential of cb-N-Apt17 as a promising candidate for antiviral therapy against different SARS-CoV-2 variants.

## DISCUSSION

The N protein of SARS-CoV-2 plays a crucial role in virus assembly and replication by forming LLPS (6, 17–20). Inhibition of N protein LLPS has been shown to inhibit SARS-CoV-2 replication (18). In our study, we confirmed the formation of N protein LLPS, and we selected a single-stranded DNA aptamer called N-Apt17 that effectively inhibits N protein LLPS.

Aptamers are short oligonucleotide sequences generated through the SELEX process, which allows them to fold into tertiary structures and specifically recognize target molecules, similar to antibodies recognizing antigens (21). Aptamers have several advantages over antibodies in clinical applications. They can be easily synthesized and modified *in vitro*, are nonimmunogenic *in vivo*, and have low molecular weight, enabling rapid tissue penetration and clearance from the bloodstream. Consequently, aptamers have gained attention for clinical diagnosis and therapy (22–24).

While previous studies have primarily focused on aptamers targeting the SARS-CoV-2 spike protein (25–27), which is susceptible to mutations and variant escape, we aimed to investigate aptamers targeting the more conserved and stable N protein (28). In contrast to the SARS-CoV-2 spike protein, the N protein demonstrates a higher degree of conservation and stability, with a remarkable 91% and 49% similarity to the protein sequences of SARS-CoV-1 and MERS-CoV, respectively (29, 30). Moreover, when considering the similarity not only in protein sequence but also in secondary structure, the analysis of the functional domain structures of the SARS-CoV-2 N protein in comparison to those of SARS-CoV-1 and MERS-CoV reveals notable similarities among these three coronaviruses (31).

In our study, N-Apt17 exhibited significant inhibition of N protein LLPS among the six selected aptamers. For successful *in vivo* applications, stability against degradation in the body is a prerequisite for aptamers. To improve the *in vivo* stability of N-Apt17, we designed and constructed a circular bivalent aptamer, cb-N-Apt17. In comparison to N-Apt17, cb-N-Apt17 showcased superior stability, enhanced binding affinity with the N protein, and maintained inhibitory effects on N protein LLPS. Additionally, cb-N-Apt17 demonstrated heightened binding affinity and specificity with major SARS-CoV-2 N protein variants, effectively impeding the viral replication of SARS-CoV-2 GFP/ΔN trVLP.

Certainly, it is noteworthy that circular bivalent aptamers (cb-N-Apt17), despite enhancing resistance against nucleases, necessitate further modifications to improve their *in vivo* half-life for prospective clinical applications. Additionally, a comprehensive assessment of the impact of this class of small molecule DNA on overall cell metabolism and gene expression is essential to ensure its safety for clinical use.

In summary, through the SELEX process, we identified N-Apt17, a high-affinity ssDNA aptamer capable of binding to the N protein and inhibiting viral replication. Our findings suggest that N-Apt17 holds promise as a potential therapeutic option for SARS-CoV-2 treatment, providing a novel approach for the prevention and management of COVID-19.

## MATERIALS AND METHODS

### Chemicals and reagents

The ssDNA oligonucleotides and SARS-CoV-2 nucleocapsid protein coding sequences used in this study were synthesized by Beijing Aoke Dingsheng Biotechnology Co., Ltd. The specific sequences of the ssDNA oligonucleotides are provided in Table S1. The following reagents and kits were purchased from the respective suppliers: isopropyl β-D-thiogalactoside (IPTG), kanamycin, Lipo293 Transfection Reagent, Bradford Protein Concentration Determination Kit, the His-tag Purification Resin, Alexa Fluor 555-labeled Donkey Anti-Rabbit (H + L) antibody, Anti-Flag Magnetic Beads, and Anti-His Magnetic Beads were obtained from Beyotime Co., Ltd. Taq DNA polymerase, T4 DNA ligase, and CCK-8 Cell Counting Kit were purchased from Vazyme Biotechnology Co., Ltd. Anti-Flag Monoclonal Antibody, Anti-His Monoclonal Antibody, and anti-GAPDH Monoclonal Antibody were purchased from Proteintech Group, Inc. Dulbecco’s modified Eagle’s medium (DMEM; high glucose) and penicillin-streptomycin (liquid) were obtained from Thermo Fisher Scientific, Inc.

### Plasmids, cells, and transfection

Sequences encoding N protein of the SARS-CoV-2 original strain (GeneBank no. MN908947.3), delta strain (GeneBank no. OP801647.1), and omicron strain (GeneBank no. OQ244249.1) were chemically synthesized and amplified by PCR and then cloned into the pET28a, pCDNA3.1–3XFLAG, or pEGFP-N1 vectors. The integrity of all constructs was verified by DNA sequencing. The *E. coli* host strain BL21(DE3) was maintained and cultured in LB medium (Luria-Bertani medium) as a routine practice in our laboratory. HEK293T and Caco2 cells were obtained from the American Type Culture Collection and cultured at 37°C with 5% CO_2_ in DMEM supplemented with 10% fetal bovine serum, 100 U/mL penicillin, and 100 U/mL streptomycin. For transfection of plasmids or ssDNA aptamers into cells, Lipo2000 Transfection Reagent was used following the manufacturer’s protocol.

### Production of trVLPs

The system for the production of trVLPs was developed following a previously reported method (23). In brief, the full-length SARS-CoV-2 GFP/ΔN cDNA was used as the template for *in vitro* transcription to generate viral RNA genomes. Subsequently, the viral genomic RNAs were introduced into Caco2 cells overexpressing the SARS-CoV-2 N protein via electroporation. After 48 or 72 hours, the supernatant, which contained the trVLPs, was collected and filtered to remove cellular debris.

### RNA extract and QRT-PCR

To quantify the copies of SARS-CoV-2 trVLPs present in the cell supernatant or within cells, Trizol reagent (Invitrogen, Carlsbad, CA) was employed for the extraction of viral RNA. Real-time PCR was conducted under the following conditions: initial denaturation at 95°C for 30 seconds, followed by 40 cycles at 95°C for 5 seconds and 60°C for 30 seconds. Subsequently, a melting curve analysis was performed with a temperature range of 65°C–95°C, using a heating rate of 0.5°C/s, while continuously measuring the fluorescence.

Standard curves were generated using a plasmid containing the SARS-CoV-2 M coding gene, and these curves were utilized to calculate the total viral copies based on the Ct (Cycle threshold) values obtained from the real-time PCR analysis.

### Purification of recombinant SARS-COV-2 nucleocapsid protein

The *E. coli* BL21 (DE3) strain was transformed with the pET28a-N plasmid and induced with 1 mM IPTG overnight at 37°C. The bacteria were subsequently harvested by centrifugation and then subjected to sonication for cell disruption. The resulting lysate was centrifuged at 4°C for 15 minutes at 20,000 × *g* to separate the soluble fraction. The His_6_-tagged recombinant N protein was purified from the soluble fraction using the His-tag Purification Resin following the manufacturer’s protocol.

### Selection of DNA aptamers for SARS-COV-2 nucleocapsid protein

The SELEX processes were conducted using a combination of magnetic bead-based methods, as previously reported (15). In brief, the DNA library was diluted with selection buffer [1× SB: 50 mM HEPES, 6 mM KCl, 150 mM NaCl, 2.5 mM CaCl2, 2.5 mM MgCl2, 0.01% (vol/vol) Tween-20, pH 7.4] and heated at 90°C for 3 minutes, followed by annealing at room temperature for 10 minutes. Subsequently, the N protein-conjugated magnetic beads were washed twice with 1× SB and mixed with the DNA library at room temperature for 30 minutes. After three washes with 1× SB, the magnetic beads were resuspended in 1× Taq buffer [50 mM KCl, 10 mM Tris–HCl, 1.5 mM MgCl2, 1% (vol/vol) Triton X-100, pH 9.0] and heated at 90°C for 10 minutes. The DNA in the supernatant was collected, followed by the addition of the forward primer (FP1), the reverse primer (RP1), and standard PCR amplification (PCR1). Subsequently, the PCR1 product was used as the template and amplified by PCR2 using primer FP2 and RP2. Following amplification, the ssDNA oligonucleotide was purified from the PCR2 product by 10% (wt/vol) denaturing polyacrylamide gel electrophoresis with 8 M urea. A total of 10 rounds of bead-based SELEX were performed. The selected DNA libraries were then amplified by PCR using primers with sequencing tags and subsequently analyzed using the MiSeq (Illumina) sequencing platform. The primer sequences utilized in the PCR are provided in Table S1.

### Cell viability assay

HEK293T cells were seeded at a density of 1 × 10^4^ cells per well in 96-well plates. After 24 hours of culture, various concentrations of N-Apt17 and cb-N-Apt17 were introduced. Following an additional 24 hours of incubation, CCK8 reagent was applied. Subsequently, optical density at 450 nm was measured using the BioTek Synergy HT multifunctional microplate detection system (USA). The cell viability across different wells and the determination of IC50 values for N-Apt17 and cb-N-Apt17 were subjected to statistical analysis using GraphPad Prism 9.

#### *In vitro* phase separation assay

The recombinant N proteins were dissolved in a phase separation buffer with the following composition: pH 7.0, 15 mM NaCl, 20 mM Tris–HCl, 135 mM KCl, 5 mM phosphate, 1.5 mM MgCl2, and 1 mg/mL BSA (Bovine Serum Albumin). The protein solution was then loaded onto a glass slide. Subsequently, the glass slides were covered with a cover slip and subjected to imaging using DIC microscopy (Olympus IX73).

### LLPS inhibition assay

HEK293T cells overexpressing N-GFP were transfected with FAM- or Cy5-labeled N-aptamers, including N-Apt17, N-Apt33, N-Apt36, N-Apt44, N-Apt45, N-Apt58, and cb-N-Apt17. The transfected cells were then imaged using either Olympus IX73 inverted microscopy or Olympus FV3000RS laser confocal microscopy.

### Confocal microscopy

HEK293T cells overexpressing N-3XFlag were first washed with 1× phosphate-buffered saline. Subsequently, the cells were fixed with 4% paraformaldehyde for 10 minutes and permeabilized with 0.2% Triton X-100. To prevent nonspecific binding, the cells were blocked with 3% BSA. The appropriate primary antibody, such as anti-Flag monoclonal antibody, was then applied to the cells, followed by incubation with a fluorescent dye-conjugated secondary antibody (such as Alexa Fluor 555 labeled).

To visualize the interaction between the N-3XFlag protein and the N-aptamers, the cells were further incubated with FAM-labeled N-aptamers (specifically N-Apt17, N-Apt33, N-Apt36, N-Apt44, N-Apt45, and N-Apt58) at room temperature for 30 minutes. After this incubation period, the cells were imaged using Olympus FV3000RS laser confocal microscopy.

### Flow cytometry

In order to immobilize His_6_- or Flag-tagged proteins, either anti-His magnetic beads or anti-Flag magnetic beads were utilized. The N-aptamers were added and incubated with the immobilized proteins in a binding buffer (1× SB) for 30 minutes at room temperature. Subsequently, the magnetic beads were washed three times to remove any unbound or nonspecifically bound molecules. The samples were then analyzed using flow cytometry (Beckman Coulter, USA).

### MicroScale Thermophoresis

The MST-based *in vitro* binding assay was conducted using a Monolith NT.115 instrument (NanoTemper Technologies) equipped with blue/red filters. FAM- or Cy5-labeled N-aptamers, which were diluted by half and half, were mixed with N protein in a buffer consisting of 20 mM HEPES (pH 7.5) and 1% Tween-20. The concentration range covered spanned from 2 nM to 100 µM. The sample was then loaded into Monolith standard-treated capillaries (NanoTemper Technologies), and the assay was performed at 25°C.

During the assay, the laser power was set to 30% with a 30-second on-time. All binding experiments were repeated three times for each measurement to ensure reliability. The acquired data were subsequently analyzed using the NanoTemper analysis software provided by NanoTemper Technologies.

### Construction of circular bivalent aptamers

The HPLC-purified DNA library and ssDNA aptamers listed in Table S1 were synthesized by Beijing Aoke Dingsheng Biotechnology Co., Ltd. To prepare the circular bivalent N-Apt17 aptamer (cb-N-Apt17), its two components (N-Apt17-1 and N-Apt17-2) were dissolved in T4 DNA ligase buffer at appropriate concentrations. The mixture was then heated at 95°C for 5 minutes, followed by rapid chilling to 16°C. Subsequently, the mixture was incubated with T4 DNA ligase at 16°C for 12 hours to facilitate the formation of the circular bivalent aptamer, cb-N-Apt17. Afterward, the solution was heated to 75°C for 10 minutes to denature the T4 DNA ligase.

The successful construction of the divalent aptamer was analyzed by agarose electrophoresis. Prior to usage, the concentration of DNA was determined using UV-vis spectrophotometry.

### Stability analysis of aptamers in serum and cells

N-Apt17 or cb-N-Apt17 were incubated in RPMI 1640 medium supplemented with 10% FBS at 37°C. At specific time points, the samples were stored at −80°C until all the desired time point samples were collected. Subsequently, the samples were thawed on ice and subjected to agarose electrophoresis.

For Cy5-labeled N-Apt17 or Cy5-labeled cb-N-Apt17, they were transfected into HEK293T cells individually. At the designated time points, the Cy5 fluorescence signal intensities were observed using Olympus FV3000RS laser confocal microscopy. This allowed for the visualization and analysis of the intracellular localization and dynamics of the Cy5-labeled aptamers over time.

### *In vivo* fluorescence imaging

Six-week-old C57/BL6 mice were anesthetized with a combination of tranquilizer and anesthetic to ensure immobilization. Subsequently, Cy5-labeled cb-N-Apt17 or Cy5-labeled N-Apt17 was injected intravenously via the tail vein. At the specified time points, fluorescence images of live mice were captured using an IVIS Lumina II *in vivo* imaging system. This allowed for the visualization and monitoring of the biodistribution and accumulation of the Cy5-labeled aptamers in real time within the live animals.

### Statistical analysis

The data analysis was performed using GraphPad Prism 9 software. To determine statistical significance, Student’s *t*-tests were conducted. The results were expressed as mean ± standard deviation based on three independent experiments. *P*-values less than 0.05 were considered statistically significant.

## SUPPLEMENTAL MATERIAL

Supplemental material spectrum.03410-23-s0001.docx Table S1; Fig. S1 and S2.

ASM does not own the copyrights to Supplemental Material that may be linked to, or accessed through, an article. The authors have granted ASM a non-exclusive, world-wide license to publish the Supplemental Material files. Please contact the corresponding author directly for reuse.
