# Supplementary material for: Inhibition of SARS-CoV-2 replication by a ssDNA aptamer targeting the nucleocapsid protein
Source: Microbiol Spectr. 2024 Feb 20;12(4):e03410-23. doi: 10.1128/spectrum.03410-23 (PMC10986557; doi:10.1128/spectrum.03410-23)
Supplement: Supplemental material — Table S1; Fig. S1 and S2. [file spectrum.03410-23-s0001.docx]

**SUPPLEMENTARY MATERIALS**

**Inhibition of SARS-CoV-2 replication by a ssDNA Aptamer Targeting the Nucleocapsid Protein**

Yanping Huang^a,b,1^, Congcong Huang^a,b,1^, Junkai Chen^a,b^, Siwei Chen^a,b^, Bei Li^a,b,c^, Jian Li^a,b,c^, Zhixiong Jin^a,b,c^, Qiwei Zhang^e^, Pan Pan^d*^, Weixing Du^a^*, Long Liu^a,b,c*^, Zhixin Liu^a,b,c^*

^a^ Department of Infectious Diseases, Renmin Hospital, School of Basic Medical Sciences, Hubei University of Medicine, Shiyan, China

^b^ Institute of Virology, Hubei University of Medicine, Shiyan, China

^c^ Hubei Key Laboratory of Embryonic Stem Cell Research, Hubei University of Medicine, Shiyan, China

^d^ The First Affiliated Hospital of Jinan University, Guangzhou, China

^e^ Guangdong Provincial Key Laboratory of Virology, Institute of Medical Microbiology, Jinan University, Guangzhou, China

* Yanping Huang and Congcong Huang contributed equally to this article. Author order was determined by contribution.

Address correspondence to lzx20022456@126.com (Z.X. Liu) or liulong2015@outlook.com (L. Liu) or duwx-025@163.com (W.X. Du) or panpan@jnu.edu.cn (P. Pan)

**Running title**: A DNA Aptamer Inhibits SARS-CoV-2 Replication.

**Table S1. DNA oligos used in this research**

| **Name** | **Strand components (5’ – 3’)** | **Design purpose** |
| --- | --- | --- |
| **FP1** | FAM-GCT GGA TGT TCA TGC TGG CAA A | SELEX |
| **RP1** | GCT GGA TGT GTC AAA GTA A | SELEX |
| **RP2** | TTT TTT TTT TTT TTT TTT TT-isp18-GCT GGA TGT GTC AAA GTA A | SELEX |
| **DNA library** | GCT GGA TGT TCA TGC TGG CAA A-N_40_-GCT GGA TGT GTC AAA GTA A | SELEX |
| **N-Apt17** | GCT GGA TGT TCA TGC TGG CAA AAG GTG TCA CTC CAT TCC TTA GGG GCA CCG GAA GCA TCT CTT TAC TTT GAC ACA TCC AGC | N Protein Binding |
| **N Apt 17 Cy5-1** | p-CGT AAA TCA GTC AGC TGG ATG TTC ATG CTG GCA AAA GGT GTC ACT CCA TTC CTT AGG GGC ACC GGA AGC ATC TCT TTA CTT TGA CAC AT(Cy5)C CAG C | Construction of cb-N-Apt17 |
| **N Apt 17 Cy5-2** | p-TGA CTG ATT TAC GGC TGG ATG TTC ATG CTG GCA AAA GGT GTC ACT CCA TTC CTT AGG GGC ACC GGA AGC ATC TCT TTA CTT TGA CAC AT(Cy5)C CAG C | Construction of cb-N-Apt17 |
| **SARS-CoV-2**  **M F** | GTG CCA CTC CAT GGC ACT AT | To quantify viral copies |
| **SARS-CoV-2**  **M R** | TCC TTG ATG TCA CAG CGT CC | To quantify viral copies |


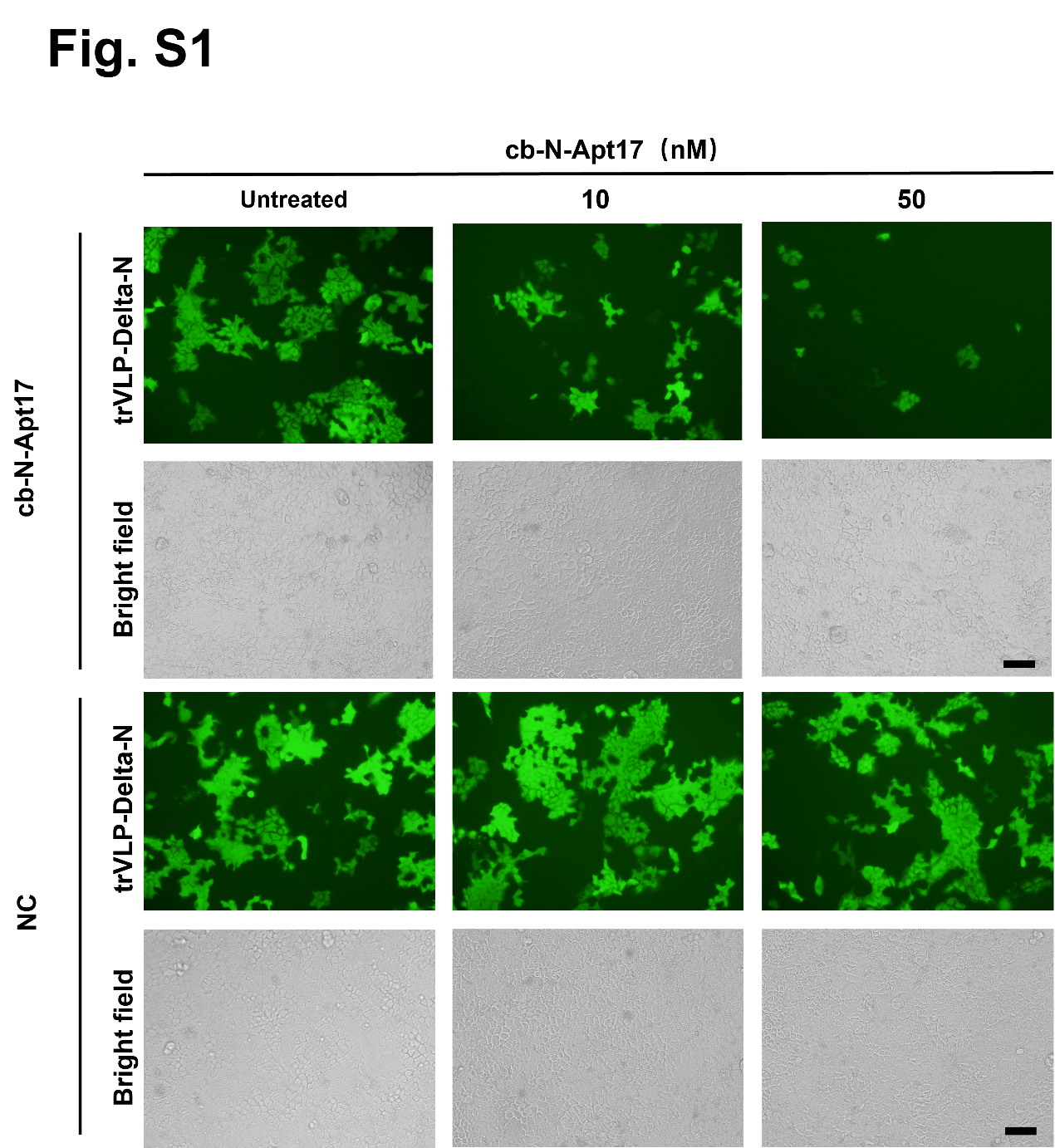


**Fig. S1** cb-N-Apt17 inhibits the replication of SARS-CoV-2 trVLP-Delta-N. Inhibition of trVLP-Delta-N infection (0.5 MOI) by cb-N-Apt17 in Caco2 cells. Different concentrations of cb-N-Apt17 (0nM, 10nM, 50nM) were transfected into Caco-2 cells that overexpressed N-Delta (Delta variant N protein). Subsequently, microscopic images were captured after infecting the cells with tr-VLP for a duration of 12 hours.


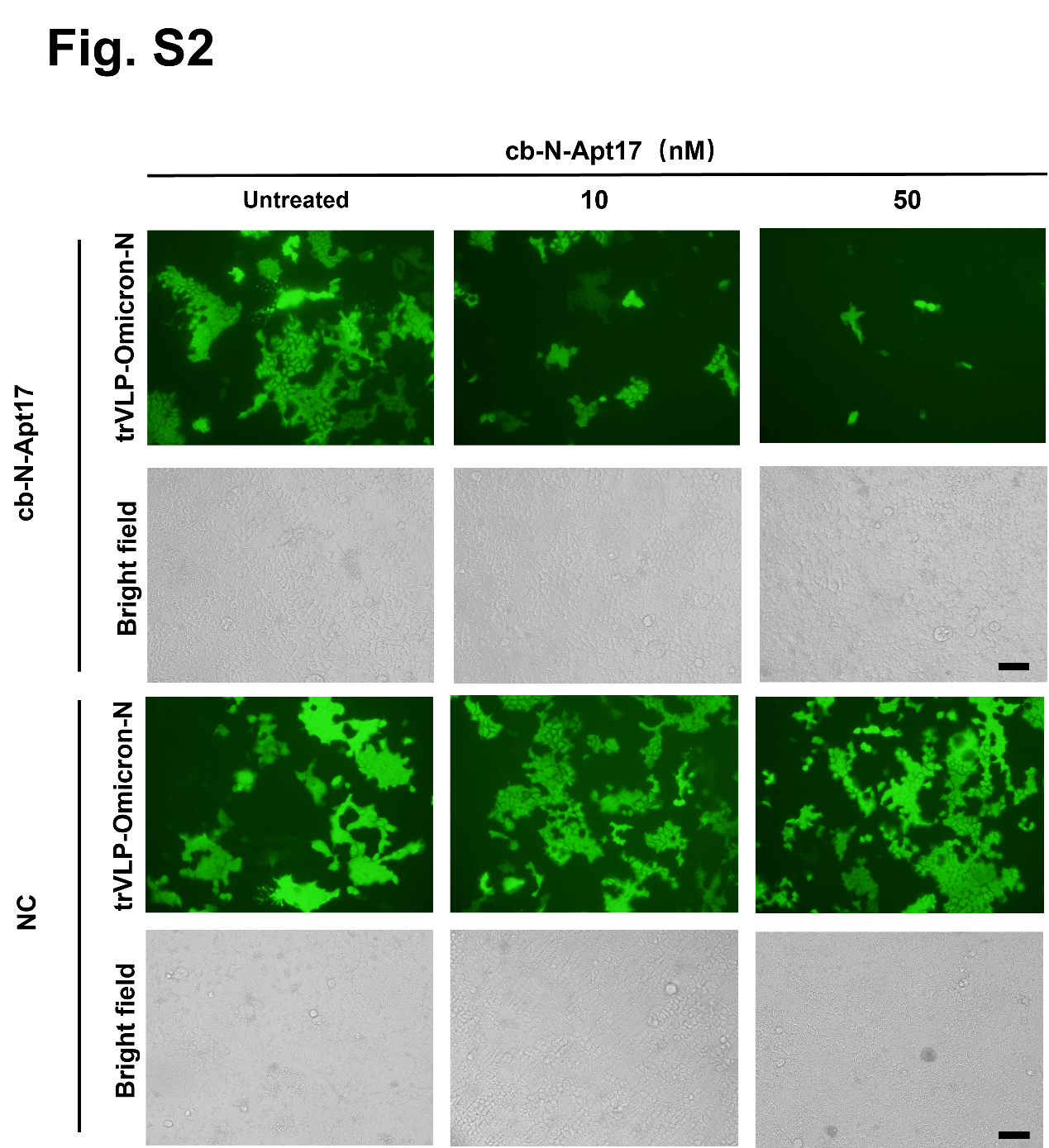


**Fig. S2 cb-N-Apt17 inhibits the replication of SARS-CoV-2 trVLP-Omicron-N.** Inhibition of trVLP-Omicron-N infection (0.5 MOI) by cb-N-Apt17 in Caco2 cells. Different concentrations of cb-N-Apt17 (0nM, 10nM, 50nM) were transfected into Caco-2 cells that overexpressed N-Omicron (Omicron variant N protein). Subsequently, microscopic images were captured after infecting the cells with tr-VLP for a duration of 12 hours.
